# Markers of tissue damage and inflammation after robotic and abdominal hysterectomy in early endometrial cancer: a randomised controlled trial

**DOI:** 10.1038/s41598-020-64016-1

**Published:** 2020-04-29

**Authors:** Evelyn Serreyn Lundin, Ninnie Borendal Wodlin, Lena Nilsson, Elvar Theodorsson, Jan Ernerudh, Preben Kjølhede

**Affiliations:** 1 Department of Obstetrics and Gynaecology in Linköping, and Department of Biomedical and Clinical Sciences, Linköping University, Linköping, Sweden; 2 Department of Anaesthesiology and Intensive Care in Linköping and Department of Biomedical and Clinical Sciences, Linköping University, Linköping, Sweden; 3 Department of Clinical Chemistry, and Department of Biomedical and Clinical Sciences, Linköping University, Linköping, Sweden; 4 Department of Clinical Immunology and Transfusion Medicine, and Department of Biomedical and Clinical and Sciences, Linköping University, Linköping, Sweden

**Keywords:** Surgical oncology, Kinases

## Abstract

The aim of this study was to analyse the dynamics of tissue damage and inflammatory response markers perioperatively and whether these differ between women operated with robotic and abdominal hysterectomy in treating early-stage endometrial cancer. At a Swedish university hospital fifty women with early-stage low-risk endometrial cancer were allocated to robotic or abdominal hysterectomy in a randomiszed controlled trial. Blood samples reflecting inflammatory responses (high sensitivity CRP, white blood cells (WBC), thrombocytes, IL-6, cortisol) and tissue damage (creatine kinase (CK), high-mobility group box 1 protein (HMGB1)) were collected one week preoperatively, just before surgery, postoperatively at two, 24 and 48 hours, and one and six weeks postoperatively. High sensitivity CRP (*p* = 0.03), WBC (*p* < 0.01), IL-6 (*p* = 0.03) and CK (*p* = 0.03) were significantly lower in the robotic group, but fast transitory. Cortisol returned to baseline two hours after robotic hysterectomy but remained elevated in the abdominal group comparable to the preoperative high levels for both groups just before surgery (*p* < 0.0001). Thrombocytes and HMGB1 were not affected by the mode of surgery. Postoperative inflammatory response and tissue damage were lower after robotic hysterectomy compared to abdominal hysterectomy. A significant remaining cortisol elevation two hours after surgery may reflect a higher stress response in the abdominal group.

## Introduction

Tissue damage and inflammatory reactions after surgery, involving several cascades of reactions, are important factors thought to affect postoperative recovery. The tissue damage and inflammatory response can be expressed and measured by changes in circulating levels of inflammatory proteins and cells, stress hormones and tissue damage markers.

The enhanced recovery after surgery (ERAS) programs aim at improving postoperative recovery without compromising the quality of care, morbidity and mortality^1,2^. The programs recommend the use of minimal invasive surgery when feasible because it is assumed to result in less tissue trauma, faster recovery and fewer postoperative complications^3,4^. The ERAS programs include measures to reduce surgical stress by afferent neural blockade with regional anaesthesia and use of carbohydrate loading preoperatively.

Some studies have shown that minimal invasive hysterectomy (vaginal or laparoscopic) results in less tissue damage with a lower inflammatory response than abdominal hysterectomy^5–7^. In addition, studies have shown significant effects on immunological response depending on the mode of hysterectomy and anaesthesia with a reduced activation of the inflammatory response and only a slight effect on cellular immunity in favour of laparoscopic hysterectomy compared with abdominal hysterectomy^8–10^. However, none of these studies were carried out using ERAS programs and thus lacked efforts to reduce surgical stress response.

Robot-assisted laparoscopy has become widespread in gynaecologic surgery since the US Food and Drug Administration’s approval of the *da Vinci*^®^, Robotic Surgery System for use in gynaecology in 2005^11^. Based on the results from previous studies of inflammatory markers after laparoscopic and open hysterectomy it could be expected that robotic surgery also should have fewer negative pathophysiological effects^8–10^. There are few randomised controlled trials published on robotic surgery in gynaecologic oncology, and only one recently published randomised trial has compared the inflammatory response and clinical recovery in robotic and abdominal hysterectomy^12^.

The present study reports secondary outcomes from our randomised controlled trial of abdominal versus robotic hysterectomy in early endometrial cancer. The primary outcome of the trial, postoperative recovery of health-related quality of life, was found to be significantly faster after robotic hysterectomy with a recovery to the preoperative level after approximately three weeks, nearly two weeks earlier than after the abdominal hysterectomy^13^.

Besides bringing about an in-depth picture of the course of inflammatory and tissue damage markers after surgery the aim of this study was to determine whether robotic hysterectomy in women with early-stage low-risk endometrial cancer in an ERAS model results in less inflammatory response and less tissue damage as measured by systemic inflammatory and tissue damage markers compared with abdominal hysterectomy.

## Methods

A prospective randomised controlled study of women with early-stage endometrial cancer, comparing robotic and abdominal hysterectomy in an ERAS program was undertaken at the Department of Obstetrics and Gynaecology at the University Hospital in Linköping, Sweden. The Regional Ethical Review Board in Linköping, Sweden approved the trial (Dnr 2011/108-31; approval date: May 19, 2011). All experiments in this trial were performed in accordance with relevant guidelines and regulations according to Swedish legislation. The study was registered in ClinicalTrial.gov Protocol Registration System (NCT 01526655) and first posted, February 6^th^, 2012 (http://clinicaltrials.gov).

Women admitted for surgical treatment of endometrial cancer, assessed by the gynaecological oncologist as International Federation of Gynaecology and Obstetrics (FIGO) stage I, low-risk endometrial cancer (endometrioid adenocarcinoma, FIGO grade 1 and 2) and scheduled for hysterectomy and bilateral salpingo-oophorectomy with peritoneal washings between February 2012 and May 2016 were asked to participate in the study. After having received informed consent, the women were randomised to either robotic or abdominal hysterectomy. Twenty-five women were randomised to abdominal hysterectomy and 25 to robotic hysterectomy. Details about the study design, flow chart, inclusion and exclusion criteria, material and methods have previously been described in details^13^.

All participants had routine preoperative evaluation, and standard pre-admission testing, and received identical information about the care and perioperative advice according to the ERAS program that was adopted at the department^14^. A strictly defined ERAS program was used including measures to reduce surgical stress by afferent neural blockade with regional anaesthesia and use of carbohydrate loading preoperatively and with a standardisation of all parts in the perioperative management using the best standard of care. Anaesthesia, postoperative analgesia and perioperative fluid therapy were likewise standardised and similar in both groups.

The abdominal hysterectomy was conducted through a transverse lower abdominal skin incision. The robotic surgery was performed with four robotic ports and three robotic arms using the *da Vinci*^®^, Surgery System. Basically, the surgery in both groups was performed according to the technique applied in minimal invasive surgery with the use of a bipolar vessel sealing device. All operations were performed by board-certified gynaecological oncology surgeons. The women received a single dose prophylactic antibiotic preoperatively, and thrombosis prophylaxis with low-molecular-weight heparin (tinzaparin 4500 IE) was given once daily for 28 days postoperatively. The postoperative care followed the ERAS program. After discharge from the hospital, the research nurse had contact with the patient regularly to collect the blood samples and to register possible complications. At the six-week end-of-study visit the patient was examined by a gynaecologist.

### Selection of inflammatory and tissue damage markers

We selected a panel of markers that has previously been shown to reflect acute inflammation and response to tissue damage after surgery and stress^15–18^. The panel consisted of high sensitivity C-reactive protein (hs-CRP), white blood cells (WBC), thrombocytes, interleukin-6 (IL-6), creatine kinase (CK), high-mobility group box 1 protein (HMGB1) and cortisol.

### Collection and analysis of blood samples – time frame

Markers of inflammatory response and tissue damage were evaluated in peripheral venous blood. Blood samples were collected on seven occasions from all women; one week preoperatively (Time 1), on the day of surgery before the operation (Time 2), postoperatively at two hours (Time 3), 24 hours (Time 4) and 48 hours (Time 5), and at one week (Time 6) and six weeks after surgery (Time 7). The samples were centrifuged within one hour after collection and the aliquots frozen at −70 °C. Analyses of the samples were carried out on one occasion, except for cell counting, which was performed immediately after the blood sample collection. The laboratories who performed the analyses were blinded for the method of intervention.

### Methods of laboratory analyses

The hs-CRP and cortisol levels were measured using a Cobas e 602 analyzer as part of a Cobas 8000 modular analysis series (Roche Diagnostics, Germany) using latex particle-enhanced immunoturbidimetric assay (‘Cardiac C-Reactive Protein (Latex) High Sensitive’ reagent kit) and Cortisol II reagents (Roche Diagnostics, Germany), respectively.

The WBC and thrombocytes were analysed by a CellDyn Sapphire Hematology Analyzer (Abbott Laboratories, Il, USA).

IL-6 was measured with MILLIPLEX^®^ MAP Kit, Human Cytokine/Chemokine Magnetic Bead Panel (Millipore Corporation, Billerica, MA, USA) on the Luminex^®^200^™^ (Invitrogen, Merelbeke, Belgium) instrument according to the manufacturer’s instructions, except that one extra standard point was added to the standard curve by one additional serial dilution. The lowest standard point was 1.6 pg/mL, and values below were assigned half of this value. Data collection was conducted using the xPONENT 3.1™ software (Luminex Corporation, Austin, TX, USA) and data analysis using the MasterPlex 2010 2.0 software (MiraiBio Group, Hitachi Solutions America, Ltd., San Francisco, CA, USA).

CK was measured using a Cobas e 701 analyzer as part of Cobas 8000 modular analysis series (Roche Diagnostics, Germany) using creatine kinase reagents from Roche.

HMGB1 was measured by HMGB1 Elisa (IBL International GMBH, Hamburg, Germany) according to the manufacturer’s instructions. The lower limit of detection was 0.1 ng/mL, and levels below (in 5% of the samples) were assigned a value of 0.05 ng/mL.

### Statistics

Sample size estimation for the study was based on the primary outcome of the study, the EQ-5D health index, and was estimated at 50 participants^13^. The sample size for secondary outcomes was based on the assumptions from earlier trials comparing laparoscopic with abdominal hysterectomy, which showed a difference in CRP between the groups of about 30 mg/mL and a standard deviation of the CRP of 16 mg/mL^5,19^. With an α = 0.05 and 1-β = 0.90, a sample size of 14 women in each group, which means a total of 28 including a drop-out of 10% was necessary to demonstrate that the groups differed significantly in CRP levels.

Nominal data were analysed by means of Chi-2 test and Fisher’s exact test. Repeated measures ANOVA models were applied to analyse continuous data measured on more than two occasions (Time 2 to Time 7). To ensure that the assumptions of the repeated measures analysis of variance (ANOVA) were met, assessment of normal distribution was performed using normal probability (Q-Q) plots and the homogeneity of variance was assessed using the Mauchly sphericity test. If the sphericity was violated and epsilon <0.75, adjustments of the within subjects factor were made with the Greenhouse-Geisser correction method; if epsilon ≥0.75 the Huynh-Feldt method was applied. Post hoc tests for between-groups were conducted using the Tukey honestly significant difference test to reveal significance between the groups on the individual occasions of sampling. As the IL-6 and HMGB1 were not normally distributed, logarithmic transformation of these variables was used. The significance level was set at *p* < 0.05 in two-tailed tests. All analyses were carried out according to intention-to-treat principles.

The software TIBCO Statistica^TM^, version 13.5 (TIBCO Software Inc, Palo Alto, CA 94303, USA) was used to carry out the statistical analyses.

## Results

**Figure 1:**
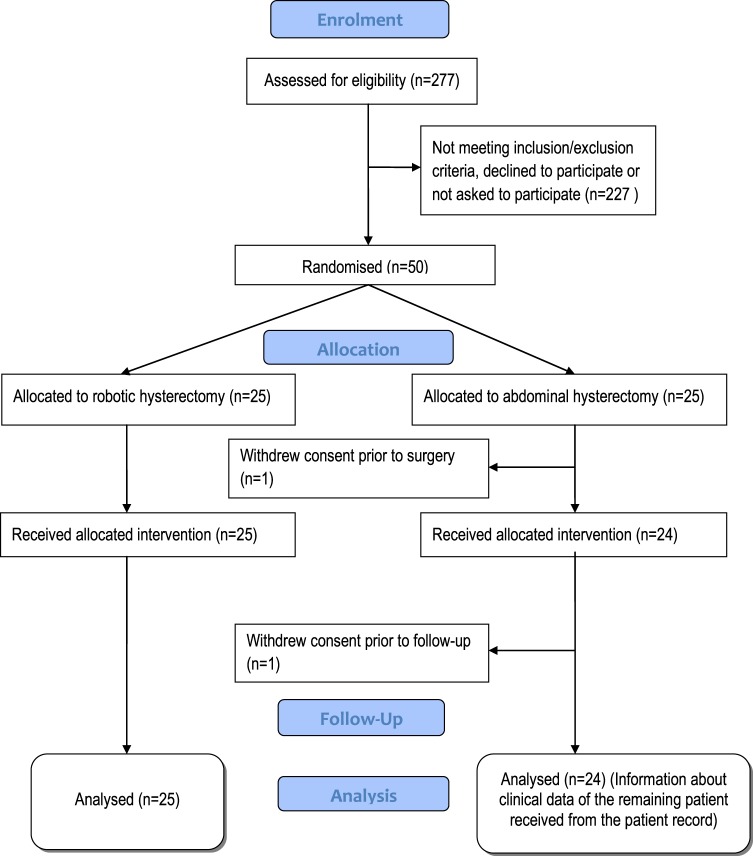
Flow chart of the participants in the study.

The flowchart of the study participants is presented in Fig. 1. Twenty-four of the 25 women allocated to abdominal and all 25 allocated to robotic hysterectomy completed the study. The demographic and clinical perioperative data are demonstrated in Table 1.

**Table 1:** Clinical and perioperative data.

Characteristics		Robotic hysterectomy (n = 25)	Abdominal hysterectomy (n = 24)
Age (years)		68 (38–83)	67 (45–85)
Body mass index (kg/m^2^)		28.2 (21.5–54.1)	28.0 (19.4–37.8)
Parity		2 (0–5)	2 (0–5)
Smokers (no. of women)		4 (16%)	0 (0%)
ASA classification:	Class I (no. of women)	9 (36%)	11 (46%)
	Class II (no. of women)	15 (60%)	12 (50%)
	Class III (no. of women)	1 (4%)	1 (4%)
Operation time (minutes)^#^		70 (48–125)	56 (41–104)
Estimated per-operative blood loss (mL)		50 (20–150)	50 (10–250)
Anaesthesia time (minutes)^#^		147 (112–239)	115 (70–177)
Adverse events during hospital stay (no. of women)^‡^		2 (8%)	5 (21%)
Adverse events after discharge (no. of women)		6 (24%)	10 (42%)
Infectious adverse events after discharge (no. of women)*		2 (8%)	5 (21%)

Figures denote median (and range) or number (and percent). ASA, American Society of Anesthesiologists risk classification.

^#^Time of surgery (p = 0.048) and anaesthesia time (*p* < 0.0001) differed significantly between the groups in the univariate analysis.

^‡^No infectious adverse events during hospital stay.

*Comprises wound - and lower urinary tract infections.

**Figure 2:**
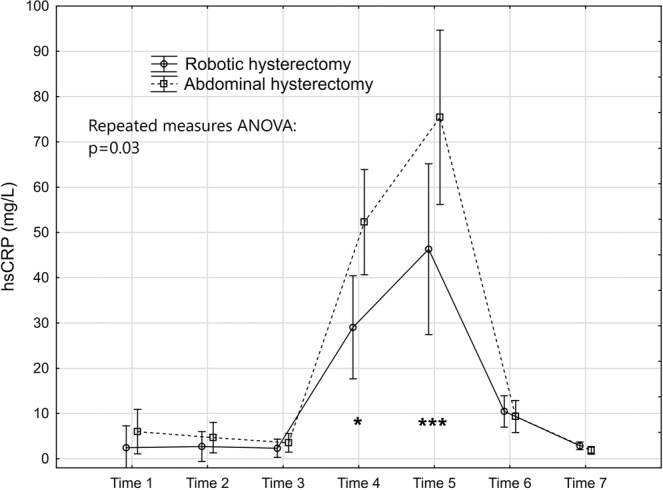
Levels and changes of high sensitivity - C-reactive protein over time. Plots indicate means and bars indicate 95% confidence interval. Time (1–7) indicates when the sample was taken in relation to time of surgery. Time 1: one week preoperatively, Time 2: on the day of surgery before the operation, Time 3: two hours postoperatively; Time 4: 24 hours postoperatively, Time 5: 48 hours postoperatively; Time 6: one week after surgery, and Time 7: six weeks after surgery. The p-value of the repeated measures ANOVA is presented and the significant post hoc tests are denoted as *(p < 0.05); ** (p < 0.01); ***(p < 0.001) or ****(p < 0.0001).

**Figure 3:**
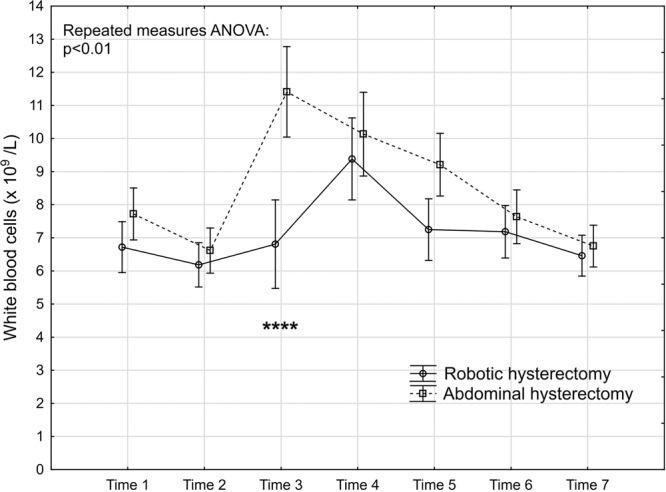
Levels and changes of white blood cells over time. For details of the figure, see legends to Fig. 2.

**Figure 4:**
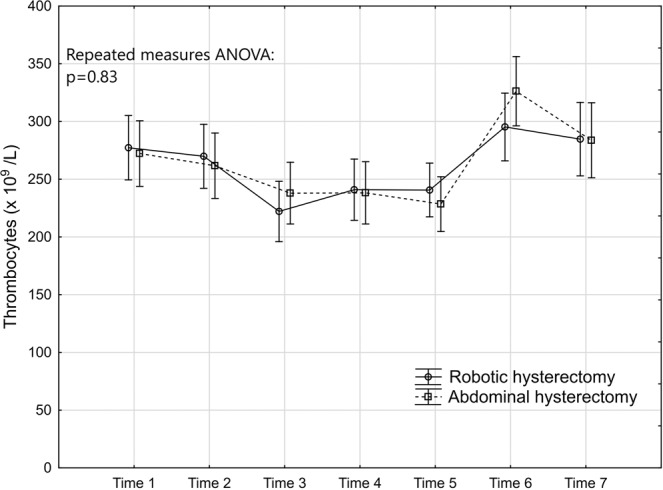
Levels and changes of thrombocytes over time. For details of the figure, see legends to Fig. 2.

**Figure 5:**
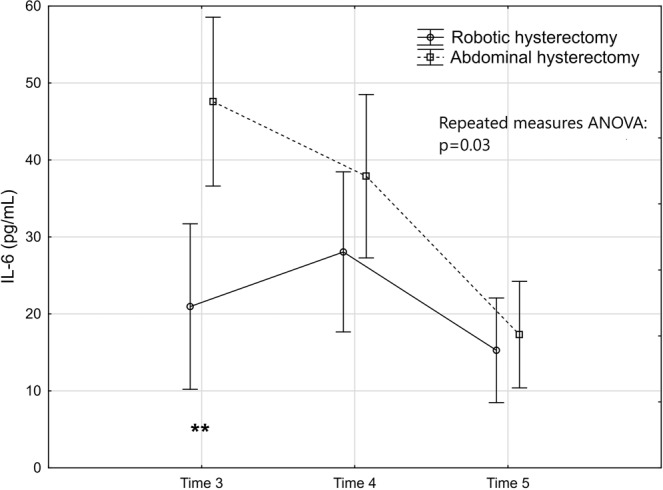
Levels and changes of interleukin-6 over time. For details of the figure, see legends to Fig. 2.

**Figure 6:**
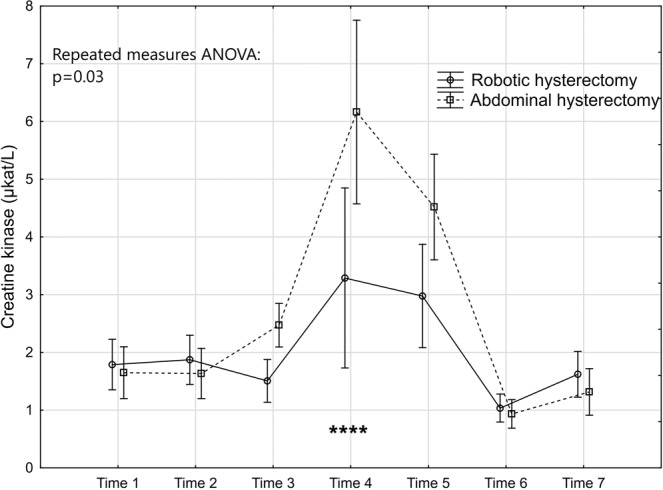
Levels and changes of creatine kinase over time. For details of the figure, see legends to Fig. 2.

**Figure 7:**
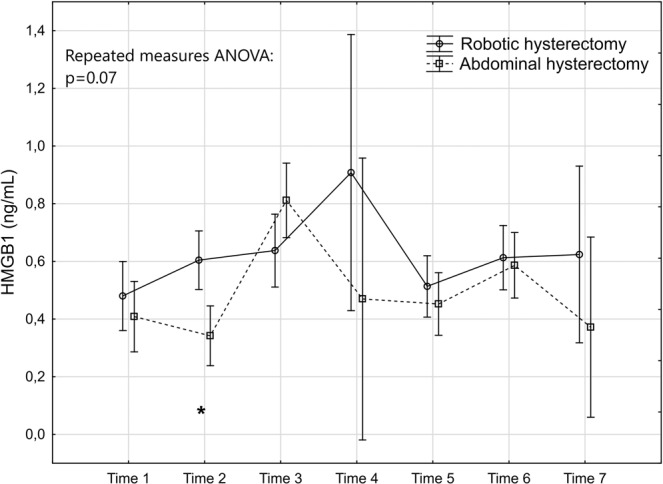
Levels and changes of high-mobility group box 1 protein over time. For details of the figure, see legends to Fig. 2.

**Figure 8:**
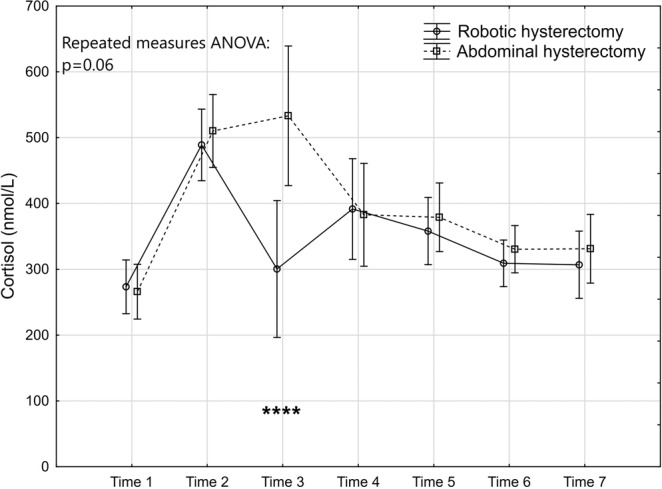
Levels and changes of cortisol over time. For details of the figure, see legends to Fig. 2.

The levels and changes over time of the seven markers are presented in Figs. 2–8.

### Inflammatory response markers

The repeated measures ANOVA (Table 2) demonstrated that hs-CRP was significantly lower in the robotic group (Fig. 2). This was mainly attributed to significantly lower levels 24 and 48 hours after the surgery. The inflammatory effect in cellular response showed a significantly lower response in WBC count in the repeated measures ANOVA in favour of the robotic group (Fig. 3). The post hoc tests revealed that the level was significantly lower than in the abdominal hysterectomy group two hours after surgery where after the levels were equalised. The thrombocytes did not seem to be influenced by mode of surgery (Fig. 4). The IL-6 levels were below the lower limit of detection (1.6 pg/mL) one week preoperatively and six weeks postoperatively (Time 1 and 7) in 43% (21/49) and in 29% (14/49) prior to surgery on the day of surgery and one week postoperatively (Time 2 and 6). At these time points, there were no significant differences between the groups, neither in the number of samples below the detection level, nor in the mean levels (Table 3). The repeated measures ANOVA comprising the levels at Time 3 to Time 5 showed a significant difference between the two groups with lower IL-6 levels in the robotic hysterectomy group (Fig. 5). This was mainly attributed to a significant difference between the groups at Time 3, as shown in the post hoc test in Table 2.

**Table 2:** Results of the repeated measures ANOVA (from Time 2 to Time 7 for all except IL-6 that was from Time 3 to Time 5) and the Tukey honestly significant difference post hoc tests for pairwise comparisons.

	Repeated measures analysis of variance (p-values)					
	Main effect between groups		Effect over time		Interaction effect	
hs-CRP	0.03		<0.0001		0.02	
WBC	<0.01		<0.0001		<0.0001	
Thrombocytes	0.83		<0.0001		<0.01	
IL-6	0.03		<0.0001		<0.001	
CK	0.03		<0.0001		<0.01	
HMGB1	0.07		<0.0001		<0.01	
Cortisol	0.06		<0.0001		<0.01	
	Tukey HSD post hoc test (p-values)*					
	**Timing**					
	**Time 2**	**Time 3**	**Time 4**	**Time 5**	**Time 6**	**Time 7**
hs-CRP			0.02	<0.001		
WBC		<0.0001				
IL-6		<0.01				
CK			<0.0001			
HMGB1	0.05					
Cortisol		<0.0001				

*Only the significant p-values in the post hoc tests are presented.

**Table 3:** Interleukin-6. Timing, number of samples below detectable level and mean level of those with detectable levels.

	Robotic hysterectomy (n = 25)	Abdominal hysterectomy (n = 24)	p-value
*Time 1:*			
Number of samples below lower detection level	10 (40%)	11 (45.8%)	0.68^#^
Mean IL-6 level of detectable samples (pg/mL)	24.3 (35.9); n = 15	19.4 (22.8); n = 13	0.72*
*Time 2:*			
Number of samples below lower detection level	5 (20%)	9 (37.5%)	0.22^§^
Mean IL-6 level of detectable samples (pg/mL)	19.4 (31.5); n = 20	14.9 (15.3); n = 15	0.97*
*Time 6:*			
Number of samples below lower detection level	6 (24%)	8 (33.3%)	0.47^#^
Mean IL-6 level of detectable samples (pg/mL)	24.0 (34.1); n = 19	14.6 (12.5); n = 16	0.65*
*Time 7:*			
Number of samples below lower detection level	10 (40%)	11 (45.9%)	0.68^#^
Mean IL-6 level of detectable samples (pg/mL)	21.2 (30.9); n = 15	21.9 (37.0); n = 13	0.91*

^#^Chi-2 test. ^§^Fisher’s exact test. *T-test performed on logarithmic transformed values.

### Tissue damage markers

The tissue damage marker CK was significantly lower in the robotic group as demonstrated in the repeated measures ANOVA (Fig. 6). This was mainly attributed to lower levels at Time 3, Time 4 and Time 5. According to the post hoc test the difference between the groups was only significant at Time 4. Concerning HMGB1, no significant difference was seen between the groups in the repeated measures ANOVA (Fig. 7).

The difference in levels of the stress marker cortisol between the groups did not reach statistical significance according to the repeated measures ANOVA (Fig. 8). However, the graphical presentation indicated that there was a difference between the groups at Time 3 (two hours after surgery) with a level in the robotic group comparable to the low level one week prior to surgery. The post hoc test confirmed the presence of a significant difference at Time 3. In contrast, the abdominal group had a sustained high level two hours after surgery comparable to the preoperative high levels for both groups (Time 2).

### Variation over time and interaction effects of inflammatory and tissue damage markers

All markers showed significant variation over time and significant interaction effects as seen in the graphs for the individual marker (Table 2). All but thrombocytes had recovered to their preoperative levels one week after surgery (Time 6). Although no significant difference was found between the groups the thrombocytes showed the highest levels on that occasion, with significantly higher levels than those seen before the operation (Time 2).

### Association between highest cortisol level and inflammatory markers

Since cortisol is a potent inflammatory and immunologic inhibitor and the cortisol levels were significantly lower in the robotic group two hours after surgery (Time 3) we analysed the potential impact of the cortisol level at that time on the outcome of the other inflammatory markers between Time 3 and Time 5 (day 2). Adding cortisol at Time 3 as a covariate in the repeated measures ANOVA models for the biomarkers revealed that cortisol was an independent factor for WBC (*p* < 0.01). Furthermore, when adjusting for cortisol at time 3, the between-group effect in WBC was no longer statistically significant (*p* = 0.06). Cortisol was otherwise not an independent factor for any of the other markers and the between-group effects shown in the repeated measures ANOVA for Time 2 to Time 7 remained significant when adjusted for Time 3 cortisol level.

## Discussion

This study showed that robotic hysterectomy in an ERAS program in early endometrial cancer resulted in a significantly lower postoperative response in inflammatory, immunological and tissue damage factors including hs-CRP, WBC, IL-6, cortisol and CK as compared with abdominal hysterectomy. The difference in the levels of the markers between the groups was of short duration. This applied in particular to the acute reacting inflammatory markers WBC, IL-6 and cortisol that were evened within the first 24 hours after surgery. The level of the markers showing delayed reaction, hs-CRP and CK, were evened within one and two days after surgery.

This trial is amongst the first describing the course of tissue damage and inflammatory stress response caused by robotic and abdominal hysterectomy in early endometrial cancer^12,15^. The strengths of this trial are the randomised design and the number and well-timed collection of blood samples on seven specified occasions in the perioperative period, giving an in-depth picture of the course of inflammatory, immunological and tissue damage markers including the use of repeated measures ANOVA tests for comparing the two modes of surgery. The use of the ERAS program including standardised anaesthesia and intrathecal morphine to keep other perioperative factors equal in the groups should also be considered as strength as it might reduce the surgical stress response.

A limitation of the study might be the sample size which was estimated based on only one of the inflammatory markers. Consequently, the results for the other markers could be underpowered when statistically insignificant. The timing of sampling was not designed to identify nadir or peak levels of the markers and thus describe the progression over time, but with the purpose to describe the levels at specific points in time in relation to the surgery. Such a construction may be seen as a limitation, in particular when a delayed response can be expected. However, all together we believe that the results of the present study are likely to be valid in patients with well-functioning hypothalamic-pituitary-adrenal (HPA)-axis and immune systems undergoing surgery in an ERAS program.

Our findings of increased inflammatory response as indicated by significantly elevated hs-CRP, WBC and IL-6 are consistent with the trial of Pilka *et al*.^15^ who prospectively observed inflammatory response and other nutritional biomarkers in patients with endometrial cancer, demonstrating a differential response to surgical trauma caused by open, laparoscopic or robotic intervention. However, Pilka *et al*. did not report for how long time the levels of markers differed or when they evened. Even the thrombocytes showed a similar pattern, although Pilka could not observe the significant thrombocytosis independent of the mode of surgery that we noticed one week postoperatively because in Pilka’s study the collection of blood samples was limited to only five days after surgery. The reactive or secondary thrombocytosis that appears after elective colorectal surgery usually reaches a peak level at a median of eight days after surgery^20^. Thus, our results might indicate that the mechanisms for developing postoperative thrombocytosis are not dependent on mode of surgery.

IL-6 is first detectable in plasma one hour after tissue trauma and stimulates the synthesis and release of C- reactive protein. IL-6, one of the most commonly measured cytokines, is a key mediator in the cascade of acute inflammatory response and has been shown to be strongly associated with the magnitude of surgical injury^16^. We were able to demonstrate this early release, which became significant between groups already two hours after surgery with a higher increase in the abdominal group, indicating more tissue damage. Our finding is according with the findings in colorectal surgery comparing inflammatory responses following robotic and open colorectal surgery^21,22^.

Chronic inflammatory diseases and postoperative infectious adverse events are factors that can influence the inflammatory biomarkers. Women with chronic inflammatory diseases were not included in the trial and the postoperative infectious complications in this trial occurred after the blood sampling one week postoperatively and had resolved at the final blood sampling after six weeks. The levels of the biomarkers were low after six weeks. It therefore seems unlikely that the infectious adverse events influenced the results significantly.

The appearance of CK in blood has generally been considered to be an indirect marker of muscle damage^23^. Earlier trials on robotic surgery and CK focused on the complication of rhabdomyolysis and found that comorbidity was more important in the development of this complication than body mass index, operating time and Trendelenburg lithotomy position^24^. Our study is the first trial evaluating CK in relation to tissue damage caused by the robotic surgery per se. We found a significantly higher level of CK after abdominal hysterectomy compared to robotic hysterectomy. Postoperatively, none of the study patients had signs of pain in the lower back, thighs or gluteal, muscle weakness of the arms or legs, or dark red or brown urine or oliguria excluding rhabdomyolysis as a cause of the elevated CK level. This implied that more muscle damage occurred with the abdominal approach. The abdominal hysterectomy was conducted through a low transverse skin incision and the rectus abdominis muscles were separated in the linea alba and lateralized gently with a self-holding retractor. In spite of this, the damage to the musculature seemed to be more extensive as measured by creatine kinase than for the robotic surgery with four 8 to 10 mm laparoscopy ports bluntly penetrating the abdominal wall musculature.

HMGB1 is an abundant nuclear protein that is passively released from necrotic or injured cells^18^. Peltz *et al*. demonstrated a markedly increased HMGB1 level within one hour of injury in trauma patients with an Injury Severity Score ≥ 15. The peak plasma elevations occurred from two to six hours postinjury, with levels remaining elevated above baseline through 136 hours after the trauma^25^. As a marker of tissue damage we noted that HMGB1 did not change significantly over time or between the two modes of surgery. This could imply that due to the use of principles for minimal invasive surgery in both modes of surgery, the overall tissue damage, independent of mode of surgery, was not sufficiently extensive to establish changes in plasma levels of the marker, or the method did not have a sufficiently low limit of detection.

The persistent high level of cortisol two hours after the abdominal surgery but not after robotic hysterectomy is an interesting finding. Wijk *et al*. also demonstrated higher cortisol levels three hours after abdominal hysterectomy compared to robotic hysterectomy but did not analyse the levels immediately preoperatively or when the levels evened^12^. Porcaro *et al*. prospectively assessed serum cortisol levels 20–30 days before surgery and four hours, one, three, five and 45 days postoperatively after robotic and open radical prostatectomy. They found a significant difference between the groups in cortisol level five days after surgery, with lower mean values postoperatively and a faster recovery to the preoperative levels in the robotic group^26^. On the other hand they found a higher cortisol level four hours after the operation in the robotic group and explained this by the surgical injury and stretching of the peritoneum by the carbon dioxide in robotics, contrary to our findings. This effect was probably eliminated in our study by the use of intrathecal morphine and bupivacaine analgesia before the general anaesthesia in the ERAS model, which reduced surgical stress stimuli through the neuroendocrine pathway, resulting in lower cortisol values^27,28^. It appears in our trial that the increase from the immediate preoperative level to the level two hours after surgery in the abdominal group was insignificant. In spite of the efforts of the ERAS model in opposing surgical stress, the surgical trauma *per se* after abdominal hysterectomy might be the reason for a sustained high cortisol level two hours after surgery, which was probably induced by the effect of an increased IL-6 level on the HPA pathway. The high cortisol level two hours after the operation was an independent factor for the elevated levels of WBC but not for any of the other markers. Maybe this brief elevation of cortisol two hours after abdominal surgery was not sufficient to influence the other inflammatory and tissue trauma markers such as hs-CRP, thrombocytes, IL-6, CK and HMGB1. The association between high cortisol levels and high WBC has previously been described^29^. Evidently, the short-term high concentrations of cortisol, as observed in this trial, seem to contribute substantially more to the increase in WBC than the surgical mode per se since the significant between-group effect disappeared when adjusting for cortisol.

## Conclusion

The study showed the dynamics of inflammatory and tissue damage markers after robotic and abdominal surgery. It also confirmed that robotic hysterectomy in an ERAS program gives a lower inflammatory reaction, less tissue damage and a lower stress response as measured by hs-CRP, WBC, IL-6, CK and cortisol compared with abdominal hysterectomy in early endometrial cancer. Although the differences in these markers were brief, it seems that less tissue damage in the robotic group might contribute to a faster recovery of the patient-reported health-related quality of life, as we previously have demonstrated.

## Data Availability

The dataset generated and analysed in the study is available from the corresponding author on reasonable request and in accordance with Swedish legislation.
